# Sialidase inhibitor modulates gut microbiota and enhances mucosal protection in the treatment of ulcerative colitis

**DOI:** 10.1128/msystems.00194-26

**Published:** 2026-07-10

**Authors:** Yiming Zhao, Lulu Chen, Chunyan Li, Yi Xu, Jing Huang, Shuijiao Chen, Zheng Yu, Xiaowei Liu

**Affiliations:** 1 Department of Gastroenterology, Xiangya Hospital Central South University 616584, Changsha, China; 2 Human Microbiome and Health Group, Department of Microbiology, Xiangya School of Basic Medical Sciences, Central South University 614453, Changsha, China; 3 Department of Clinical Nutrition, Xiangya Hospital Central South University 159374, Changsha, China; 4 National Clinical Research Center for Geriatric Disorders, Xiangya Hospital Central South University 616686, Changsha, China; Katholieke Universiteit Leuven, Leuven, Belgium

**Keywords:** ulcerative colitis, intestinal mucus layer, metabolomics, mucus layer, gut microbiome

## Abstract

Ulcerative colitis (UC) is characterized by mucosal barrier erosion, a process exacerbated by bacterial sialidases. We investigated the therapeutic efficacy of the sialidase inhibitor (SI) in UC. In a pilot randomized clinical trial, SI intervention significantly improved clinical symptoms and endoscopic outcomes in mild-to-moderate UC patients. This improvement correlated with an enrichment of butyrate-producing taxa and beneficial metabolic pathways. In a dextran sulfate sodium-induced colitis mouse model, SI attenuated inflammation and restored mucus layer integrity, accompanied by increased expression of *Muc2* and *Tff3*. Crucially, unlike broad-spectrum antibiotics, SI preserved microbial community resilience while specifically enriching beneficial mucolytic commensals, *Akkermansia muciniphila* and *Bacteroides acidifaciens*. These findings identify SI as a promising therapeutic strategy that targets sialidase activity to reinforce the mucosal barrier and restore gut homeostasis.

**IMPORTANCE:**

The gut microbiota plays a pivotal role in maintaining mucosal integrity and intestinal homeostasis; however, dysbiosis-driven mucus layer degradation remains a hallmark of ulcerative colitis (UC). Current interventions like antibiotics often disrupt microbial diversity, exacerbating dysbiosis and failing to address mucosal thinning, which is a critical factor in UC progression. Developing strategies to reinforce the mucus barrier without compromising microbial balance is urgently needed, but such approaches remain underexplored. Our study demonstrates that sialidase inhibitors (SIs) uniquely preserve mucosal thickness by curbing microbial mucin degradation while selectively enriching beneficial taxa and butyrate-producing bacteria. Unlike antibiotics, SIs enhance mucosal protection without destabilizing microbial communities, offering a dual-action therapeutic strategy. This work bridges a critical knowledge gap, providing evidence for microbiota-targeted therapies that synergistically restore mucosal health and microbial ecology in UC.

**CLINICAL TRIALS:**

This study was registered with the Chinese Clinial Trial Registry as ChiCTR2000028767.

## INTRODUCTION

Ulcerative colitis (UC) is a recurrent and protracted colonic inflammatory disorder involving the rectum and extending proximally. The lesions are mainly confined to the mucosa and epithelial barrier. Clinical remission and endoscopic mucosal healing represent two conventional therapeutic endpoints in the treatment of UC (1). Despite advances in UC treatment, only about 40% of patients achieve clinical remission after treatment (2).

The intestinal epithelial barrier is composed of various types of epithelial cells, tight junctions, and the mucus layer. The intestinal mucus layer, composed of mucin 2 (MUC2) produced by goblet cells, is essential to maintaining the balance of the immune system (3) and as an important barrier to prevent the invasion of harmful bacteria into the host body (4). UC is associated with a decrease in mucin production, as well as a thinner mucus layer (5). It has been reported that structural weakening of the colonic mucus barrier is an early event in UC pathogenesis (6), and the reinforcement of intestinal barrier function is beneficial to UC patients (1).

Different from a single unattached mucus layer in the small intestine, the mucus layer in the colon comprises a looser and more hydrated outer layer providing a habitat for symbiotic bacteria (7). In the gastrointestinal tract, sialic acid residues are present at the termini of mucins (8). Some gut microbes can express and release sialidase to degrade the sialic acid at the end of the host mucin. The sialylation of mucin is essential to maintain intestinal mucus integrity (9). The gut microbiota can degrade and utilize glycoproteins, and glycoproteins in turn affect intestinal colonization of symbiotic microbiota (10). This kind of interaction between gut microbiota and the mucus layer is crucial for understanding the potential mechanisms of UC. It has been reported that the degradation of sialic acid by sialidase is a key step in initiating the degradation of MUC2 (9). Theoretically, preventing the mucous layer from being destroyed is helpful in the treatment of UC. However, few studies have investigated how inhibition of sialic acid hydrolysis from mucin affects the gut microbiota and host health. Therefore, we utilized a type of sialidase inhibitor to conduct a pilot randomized clinical trial on mild-to-moderate UC patients and performed an animal experiment using a mouse model with acute dextran sulfate sodium (DSS)-induced colitis. By performing multi-omics and experimental methods, we explored the effect of the sialidase inhibitor on gut microbiota and gut health.

## MATERIALS AND METHODS

### The pilot randomized clinical trial

As a pilot study, 19 eligible patients with mild-to-moderate UC were recruited at Xiangya Hospital (Changsha, China). Inclusion criteria were as follows: (i) patients aged 18–75 years, (ii) mild-to-moderate active UC patients based on modified Mayo score, and (iii) patients who understood the whole process of this research and provided written informed consent voluntarily. Exclusion criteria included the following: (i) patients using biologic agents, antibiotics, or glucocorticoids; (ii) patients who were allergic to salicylic acid; (iii) patients with renal creatinine clearance rate <60 mL/min; (iv) patients with active infection in intestine; (v) women in pregnancy or lactation; (vi) patients with diabetes; and (vii) participation in any other clinical study within 1 month prior to our screening visit. The eligible patients were randomly divided into Non-SI (*N* = 9) and SI groups (*N* = 10).

Patients in the SI group received both mesalazine (MES; 1 g qid, Ethypharm Co., Ltd, Shanghai, China) and oseltamivir (75 mg qd, Roche Co., Ltd, Shanghai, China) for 4 weeks, while those in the Non-SI group received mesalazine alone (1 g qid). The oseltamivir regimen used in this pilot study (75 mg once daily for 4 weeks) was selected based on previously established clinical experience and published safety evidence for extended-duration human use (11). DUBLIN endoscopic score, modified Mayo score, and symptom scores were evaluated by the gastroenterologist blindly. The adverse events included joint pain, systemic pain, arrhythmia, and gastrointestinal bleeding and were collected at 2, 4, and 12 weeks after intervention. This clinical trial was registered under the Chinese Clinical Trial Registry (ChiCTR2000028767, https://www.chictr.org.cn/showprojEN.html?proj=47789).

### 16S rRNA gene sequencing

DNA extraction was performed on total genomic DNA using the OMEGA Soil DNA Kit (M5635-01) (Omega Bio-Tek, Norcross, GA, USA). The resulting PCR amplicons were purified using Vazyme VAHTSTM DNA Clean Beads (Vazyme, Nanjing, China) and quantified with the Quant-iT PicoGreen dsDNA Assay Kit (Invitrogen, Carlsbad, CA, USA). Following quantification, amplicons were pooled in equal volumes, and paired-end 2 × 250 bp sequencing was conducted on the Illumina NovaSeq platform using the NovaSeq 6000 SP Reagent Kit (500 cycles). The specific operation refers to the previous article (12).

### 16S rRNA gene data analysis

The raw 16S rRNA gene sequences were quality-filtered, truncated, merged, and screened for low-quality reads, and chimeric sequences were removed as described in our previous studies (13, 14). Finally, the remaining sequences were clustered to operational taxonomic units (OTUs). To reduce false positives of obtaining data, only the OTU-mapped reads with more than 10 were retained and rarefied to the minimum read counts using the R package “picante” (15). Next, the taxonomic classification was performed according to the Silva database (version 138) (16). The Bray-Curtis distance metrics were calculated based on the relative abundance of OTUs. To explore the microbial correlations among groups, the SparCC (17) co-occurrence networks were constructed using FastSpar(18) (version 1.0) with OTUs presented based on different thresholds (FDR-adjusted *P*-value and *R*-value). To minimize false positives in identifying microbial co-occurrence relationships, only correlations with an FDR-adjusted *P*-value of less than 0.05 and an *R*-value greater than 0.8 were included in the network analysis. In addition, we performed linear discriminant analysis effect size (LEfSe) analysis (19) to determine biomarkers (OTUs) in different groups.

### Fecal metabolomic assays

The sample, stored at −80°C, was thawed on ice. A 400 μL solution (methanol = 7:3, vol/vol) containing the internal standard was added to 20 mg of the sample and vortexed for 3 min. The mixture was then sonicated in an ice bath for 10 min, followed by an additional vortex for 1 min, and subsequently placed at −20°C for 30 min. After this, the sample was centrifuged at 12,000 rpm for 10 min at 4°C. The sediment was discarded, and the supernatant was centrifuged again at 12,000 rpm for 3 min at 4°C. A 200 μL aliquot of the supernatant was then transferred for LC-MS analysis.

For the LC-MS analysis, the following conditions were set: the UPLC column used was a Waters ACQUITY UPLC BEH C18 (1.8 µm, 2.1 mm × 100 mm) maintained at 40°C, with a flow rate of 0.4 mL/min and an injection volume of 2 μL. The solvent system consisted of water (0.1% formic acid) and acetonitrile (0.1% formic acid). The column was initially eluted with 5% mobile phase B and then subjected to a linear gradient to 90% mobile phase B over 11 min, held for 1 min before returning to 5% mobile phase B within 0.1 min, and held for an additional 1.9 min to restore starting conditions.

Data acquisition was conducted in information-dependent acquisition mode using Analyst TF 1.7.1 Software (Sciex, Concord, ON, Canada). Source parameters were configured as follows: ion source gas 1 (GAS1) and gas 2 (GAS2) at 50 psi; curtain gas (CUR) at 35 psi; source temperature (TEM) at either 550°C or 450°C; declustering potential at 60 V or −60 V for positive and negative modes, respectively; and ion spray voltage floating at 5,000 V or −4,000 V for positive and negative modes, respectively. The TOF MS scan parameters included a mass range of 50–1,000 Da, an accumulation time of 200 ms, and dynamic background subtraction enabled. For product ion scans, the mass range was set from 25 to 1,000 Da, with an accumulation time of 40 ms, collision energy at 30 or −30 V in positive or negative modes, respectively, and a collision energy spread of 15. Additional parameters included resolution set to UNIT, charge state from 1 to 1, intensity of 100 cps, exclusion of isotopes within 4 Da, a mass tolerance of 50 mDa, and a maximum of 12 candidate ions monitored per cycle.

In addition, the enrichment analysis of metabolite data was performed using MetaboAnalyst (20), which is a comprehensive platform dedicated to metabolomics data analysis. The hypergeometric test was used to calculate *P*-values.

### Analysis of the relationship between gut microbes and metabolites

We utilized Mmvec (21), a neural network method inspired by deep learning, to construct a log-transformed conditional probability matrix for each pair of cross-omics features. Singular value decomposition was then applied to represent co-occurrence relationships in biplots. The model was selected based on achieving the highest accuracy across various initialization conditions for the gradient descent algorithm, while ensuring low cross-validation error and optimal model likelihood.

### Animals

In this study, 20 female BALB/c mice (3 weeks old, 12–15 g) were purchased from Hunan SJA Laboratory Animal Co., Ltd. (Changsha, China) and raised under specific pathogen-free conditions in a controlled environment (normal light-dark cycle, 22°C ± 2°C, and 40%–50% humidity). All mice were randomly assigned to four groups: Control, DSS, DSS + BSA, and DSS + SI. During the pretreatment phase, mice in the DSS + broad-spectrum antibiotic (BSA) group received broad-spectrum antibiotics in drinking water, and mice in the DSS + SI group received SI (0.3 g/L, Roche Co., Ltd, Shanghai, China) in drinking water, whereas the Control and DSS groups received sterile water. Acute colitis was then induced by administration of 2.5% DSS (Cat# 216011080; MP Biomedicals, CA, USA) in drinking water during the final 7 days of the experiment. The susceptibility of acute DSS-induced colitis was evaluated on the 7th day of modeling by histological scores, disease activity index (DAI), and length of colon measured by two senior pathologists in a blinded manner as described before (22). After the sacrifice, 0.5 cm descending colon was collected to fix in 10% neutral-buffered formalin for preparing paraffin-embedded sections for hematoxylin-eosin (HE) staining.

The detailed methods for real-time PCR were described in the supplementary material. The distal colon tissues were fixed by Carnoy’s fixation solution (60% methanol, 30% chloroform, and 10% glacial acetic acid) and embedded in paraffin, then deparaffinized and stained by AB-PAS (G1285; Solarbio, Beijing, China). Afterward, the sections were dehydrated, mounted, and imaged. The thickness of the mucus layer was measured by ImageJ software (perpendicular to the mucus layer, 15 measurements per mouse).

### Metagenomic sequencing and analysis

To study microbial function alteration, the shotgun metagenomic sequencing was carried out referred to our previous methods (23). Total microbial genomic DNA was extracted using the QIAGEN DNeasy PowerWater Kit (14900-100-NF). The extracted microbial DNA was processed to construct metagenome shotgun sequencing libraries. Each library was sequenced by the Illumina HiSeq X Ten platform (Illumina, USA) with a PE150 strategy.

To ensure data accuracy and obtain clean reads, raw sequences were processed to remove low-quality (average quality <35 or containing more than 16 unclear bases) reads using Fastp (24) (version 0.21.0) and FastUniq (25) (version 1.1.0) to eliminate duplicates in paired short DNA sequence reads in a FASTQ format. The sequences from mice were filtered out by mapping to the mouse reference genome (mm39) using Bowtie2 (26) (version 2.3.5) in sensitive mode. The microbial composition analysis was performed using MetaPhlAn (27) (version 4). Afterward, the clean reads were assembled into contigs using Megahit (28) (version 1.2.9) with default settings. The contigs greater than 500 bp were used to perform gene prediction with MetaGeneMark (29) (version 3.38). After that, we used hmmer (30) (version 3.3.1) based on the AntiFam database (31) to remove spurious genes. A gene catalog was established using mmseq2 (32) (version 13.45111) based on a minimum sequence identity of >95% and a clustering threshold of >90%. For each gene, reads from that sample were mapped using BWA (33) (version 0.7.17), and the gene abundance was calculated according to the mapped read number divided by gene length. The carbohydrate-active enzyme (CAZyme) profile was predicted according to the CAZyme database using dbCAN2 (34), which utilized Diamond (35), HMMER (36), and eCAMI (37) to predict CAZyme. To make the results accurate, only the annotations predicted by all three methods were retained according to our previous study (13). Referring to the research of Tailford et al. (38), we divided CAZymes into two groups (Mucin degradation and Others).

### Other statistical analyses

Procrustes analysis of the correlation between OTUs and metabolites was performed using the vegan package in R. All other statistical tests were conducted using the “scipy” or “statsmodels” package in Python. Most data visualization was performed using “ggplot2” in R or “matplotlib” in Python.

## RESULTS

### Sialidase inhibitor contributes to treating UC based on a randomized clinical trial

**Fig 1:**
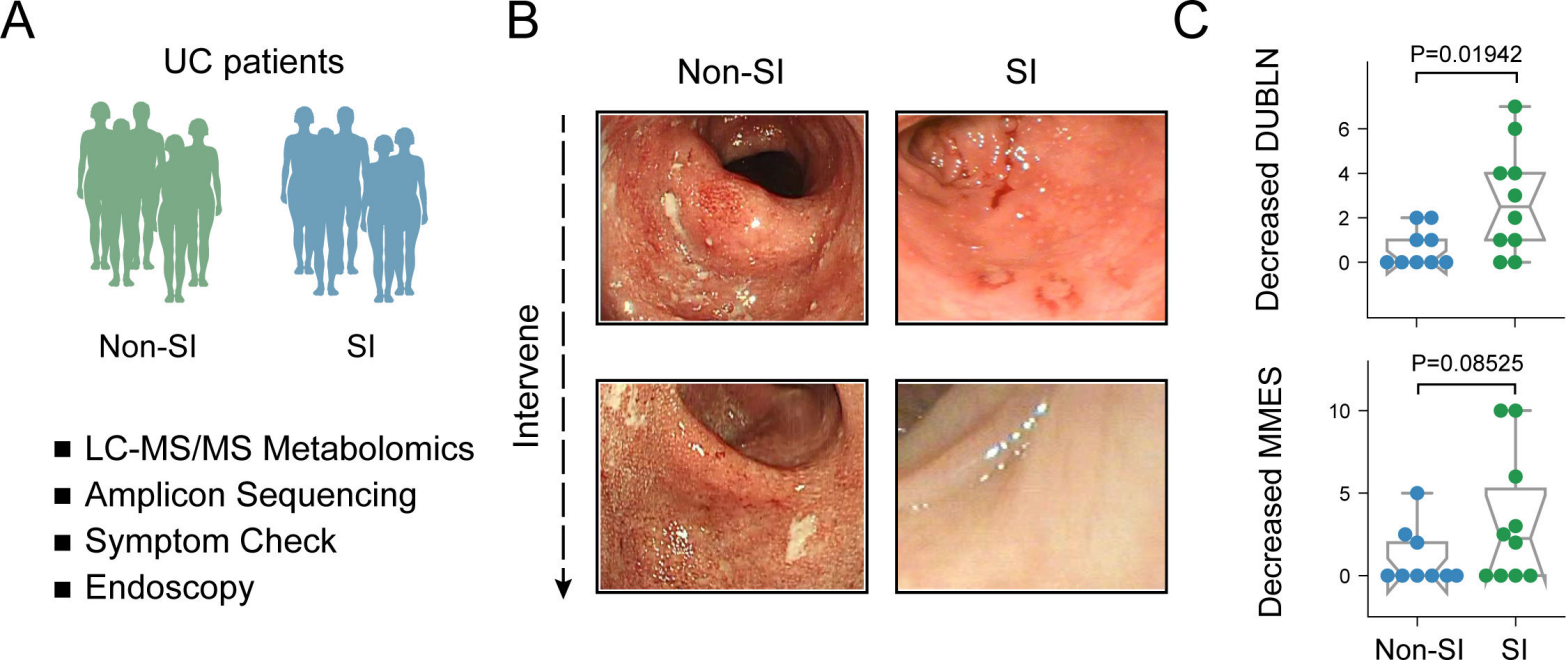
Improvement of sialidase inhibitor on the treatment of UC. (**A**) The cohorts of the Non-SI and SI groups. (**B**) The endoscopic views of two groups. (**C**) The integrated scores (DUBLIN and modified Mayo indices) are used to evaluate the therapeutic effect of the SI (Mann-Whitney and Wilcoxon test).

Between 5 June 2020 and 6 January 2022, 19 eligible mild-to-moderate UC patients were randomly assigned to receive oseltamivir (SI group, *N* = 10) or not (Non-SI group, *N* = 9, Fig. 1A). The two groups were comparable in baseline demographic and disease-related characteristics (Table S1, Fig. S1). Compared with the Non-SI group, the post-intervention improvement of abdominal pain and total symptom score in the SI group was observed (Table S2; *P*-value < 0.05). More obvious endoscopic remission was observed in the SI group after 1 month of intervention (Fig. 1B; Fig. S2). In addition, the reduction in DUBLIN score was significantly greater in the SI group than in the Non-SI group, while the modified Mayo endoscopic (MME) score showed a trend toward greater improvement in the SI group (Fig. 1C). No significant differences in adverse events were observed between the two groups (Table S3).

### Sialidase inhibitor changes the intestinal metabolic environment by altering the gut microbiota of UC patients

**Fig 2:**
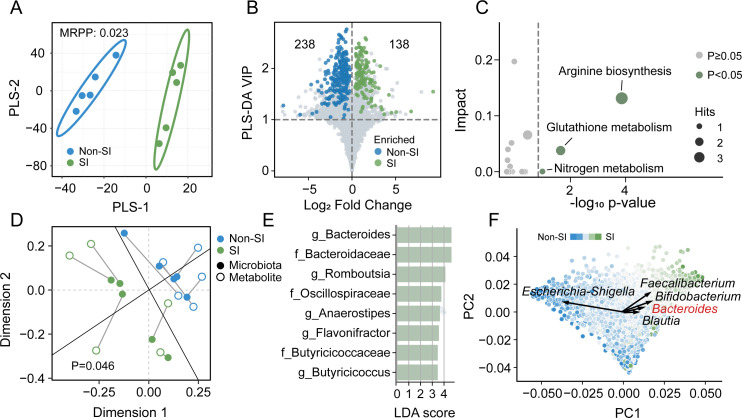
Altered gut metabolic and microbial composition after sialidase inhibitor intervention in UC patients. (**A**) The composition of metabolites in different groups with PLS-DA. The top of the figure shows the *P*-value of metabolite composition under different variables using the multi-response permutation procedure (MRPP) test. (**B**) Compared with the Non-SI group, the increasing and decreasing metabolites were shown. Only metabolites with *P*-value < 0.05 and VIP scores > 1 were colored, and metabolites were colored according to their log_2_ fold change. (**C**) The enrichment analysis of differential metabolites, and the green dots represent the pathways with *P*-value < 0.05. (**D**) Procrustes analysis of the correlation between microbial and metabolite composition (*P*-value = 0.046, 9,999 permutations). (**E**) The LEfSe analysis is used to detect different microbial biomarkers between two groups, and only the features with an LDA score of more than three are shown. No feature is detected as a biomarker in the Non-SI group. (**F**) Biplot of metagenomics and metabolic data sets using Mmvec. Metabolic features (dots) are labeled according to their strength of change with respect to fold change size (blue is associated with Non-SI, green with SI). The differential microbes were represented by arrows indicating their cooccurrences with the metabolites. Only the top five genera with the most feature importance are shown. The microbe (*Bacteroides*) which is also detected as a biomarker using LEfSe is marked in red.

Next, we detected the fecal metabolites by LC-MS/MS untargeted metabolomics to study the potential reasons why SI improves the therapeutic effect of UC. The results showed that the composition of intestinal metabolites of patients after SI intervention was significantly different from that of patients in the Non-SI group (Fig. 2A, *P*-value = 0.023). Compared with the Non-SI group, there were 376 metabolites that changed significantly after SI intervention (Fig. 2B; VIP-score > 1 and *P*-value < 0.05), which were enriched in the pathways of arginine biosynthesis, glutathione, and nitrogen metabolism (Fig. 2C). Among the differential metabolites involved in these enriched pathways, urea was significantly increased after SI intervention, whereas L-glutamate, N-acetylornithine, and 5-oxoproline were significantly decreased (Table S4).

In addition, using 16S rRNA gene sequencing and the Procrustes analysis, we found that the intestinal metabolic landscape was significantly related to gut microbiome (Fig. 2D). LEfSe analysis showed that there were several bacteria enriched in the SI group; interestingly, all of them (*Bacteroides*, *Romboutsia*, *Anaerostipes*, *Flavonifractor*, and *Butyricicoccus*) are capable of producing butyrate (Fig. 2E). Afterward, Mmvec, a bioinformatic tool utilizing deep-learning model, was performed and trained to a highly accurate level on our data for investigating metabolite-microbe associations (Fig. S3). Compared to SI, *Escherichia-Shigella*, one of the usual pathogens affecting gut health (39), was detected to be associated with the Non-SI group. In contrast, the relative abundance of beneficial gut microbiota, such as *Faecalibacterium*, *Bifidobacterium*, *Bacteroides,* and *Blautia,* were found increased after SI intervention. *Bacteroides* was also the biomarker increased in the SI group (Fig. 2F). These results emphasized the effect of SI on the relationship between the altered metabolic spectrum and gut microbiota.

### Sialidase inhibitor has a protective effect on the intestinal mucus layer

**Fig 3:**
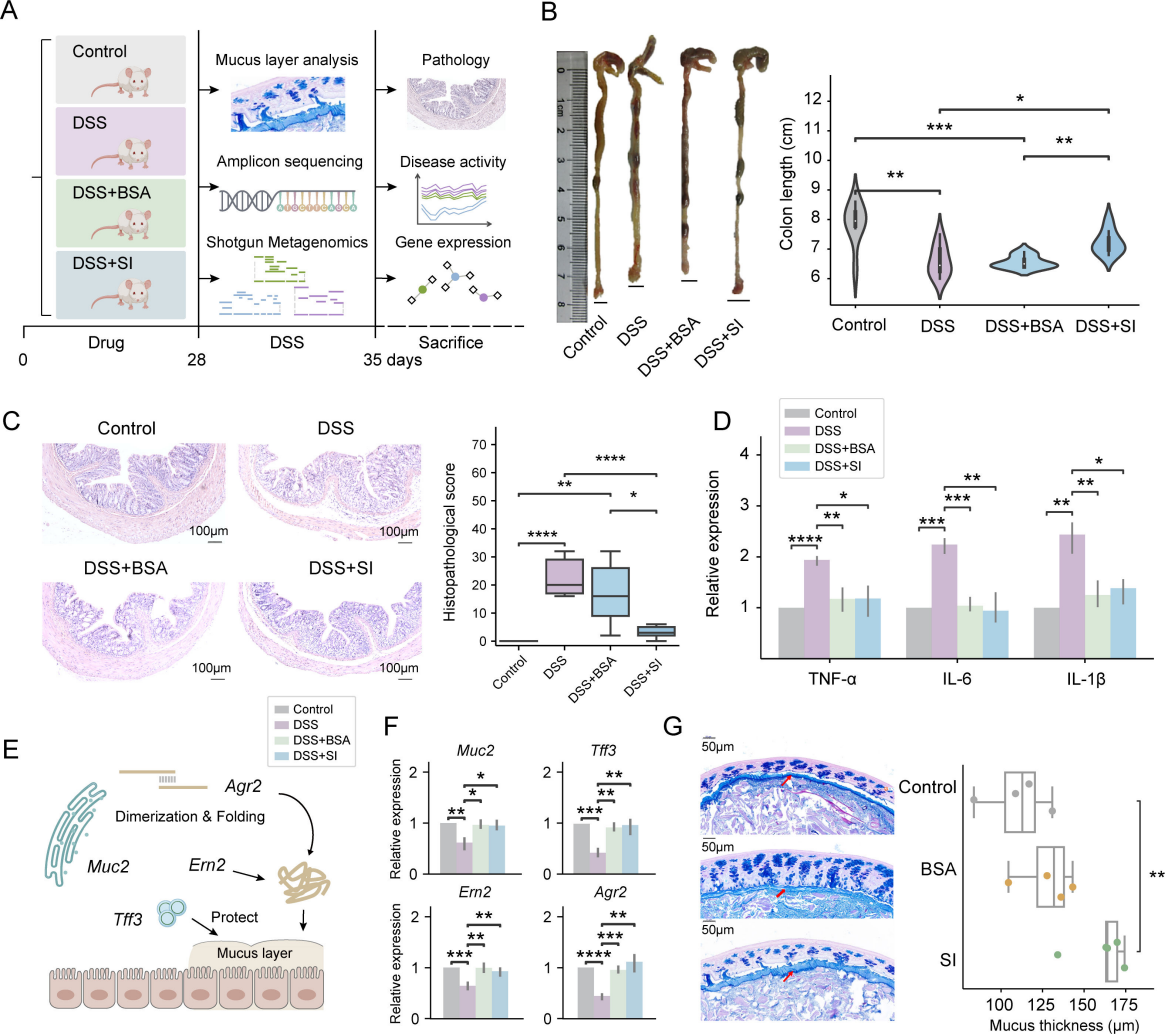
Sialidase inhibitor intervention increased the thickness of the colonic mucus layer in mice. (**A**) The scheme of the mouse research design. (**B**) Colon length of mice in different groups. (**C**) Hematoxylin-eosin (HE) staining (magnification 20×) of the colon tissues and histopathological scores in different groups. All the results are presented as mean ± standard deviation. (**D**) Relative mRNA expression of TNF-α, IL-6, and IL-1β in different groups. (**E**) Schematic diagram of the production of the intestinal mucus layer in the body. (**F**) Relative mRNA expression of MUC2, Tff3, Ern2, and Agr2 in different groups. (**G**) Alcian blue and periodic acid-Schiff staining (magnification 20×) and mucus thickness in different groups. (*0.01 < *P*-value < 0.05; **P-value < 0.01; ***P-value < 0.001; ****P-value < 0.0001, Mann-Whitney and Wilcoxon test)

To further investigate the effect of SI on intestinal inflammation, different groups of mice were set up, and the effect of the BSA and the SI was explored separately (Fig. 3A). The results demonstrated that the use of the BSA was not an effective intervention for DSS-induced colitis. However, the SI exhibited an inhibitory effect on enteritis. In detail, the SI alleviated DSS-induced shortening of colon length (Fig. 3B) and reduced DAI (Fig. S4), which was also verified by the check of pathology (Fig. 3C). Although the BSA did not protect the colon tissue effectively, the altered expression of *Tnf-*α, *IL-6*, and *IL-1*β mRNA suggested a potential function of microbiota in promoting inflammation (Fig. 3D).

The intestinal mucus layer plays a crucial role in preventing the invasion of harmful microbes into the intestinal mucosa (40). The synthesis of intestinal mucus is primarily regulated by the *MUC2* gene, which is responsible for the production of mucin (41). As key regulators of mucus production, the expression of *Ern2*, *Agr2*, and *Tff3* genes was reduced in the DSS-induced group; however, normal expression levels were maintained in SI- or BSA-treated groups, suggesting that the SI can inhibit the intestinal damage gene expression and microbial-mediated mucosal changes in the host (Fig. 3E and F). Unsurprisingly, the use of SI increased the thickness of the intestinal mucus layer. While BSA resulted in a slight increase in mucus layer thickness, its structure became less compact (Fig. 3G), potentially due to a reduction in symbiotic beneficial microbes within the mucus layer. This explains why BSA was less effective in suppressing enteritis. Owing to the fact that the mucus layer of the intestine is primarily secreted by goblet cells, we also assessed the number of goblet cells in the SI and Control groups. The results demonstrated that the SI intervention did not elevate the number of colonic goblet cells (Fig. S5). These findings suggested that the SI intervention facilitated the expression of pathways associated with normal mucus secretion in the organism and that the thicker intestinal mucosal layer enabled the host to combat intestinal inflammation.

### Sialidase inhibitor promotes the enrichment of beneficial gut microbiota on the premise of ensuring the stability of microbial community

**Fig 4:**
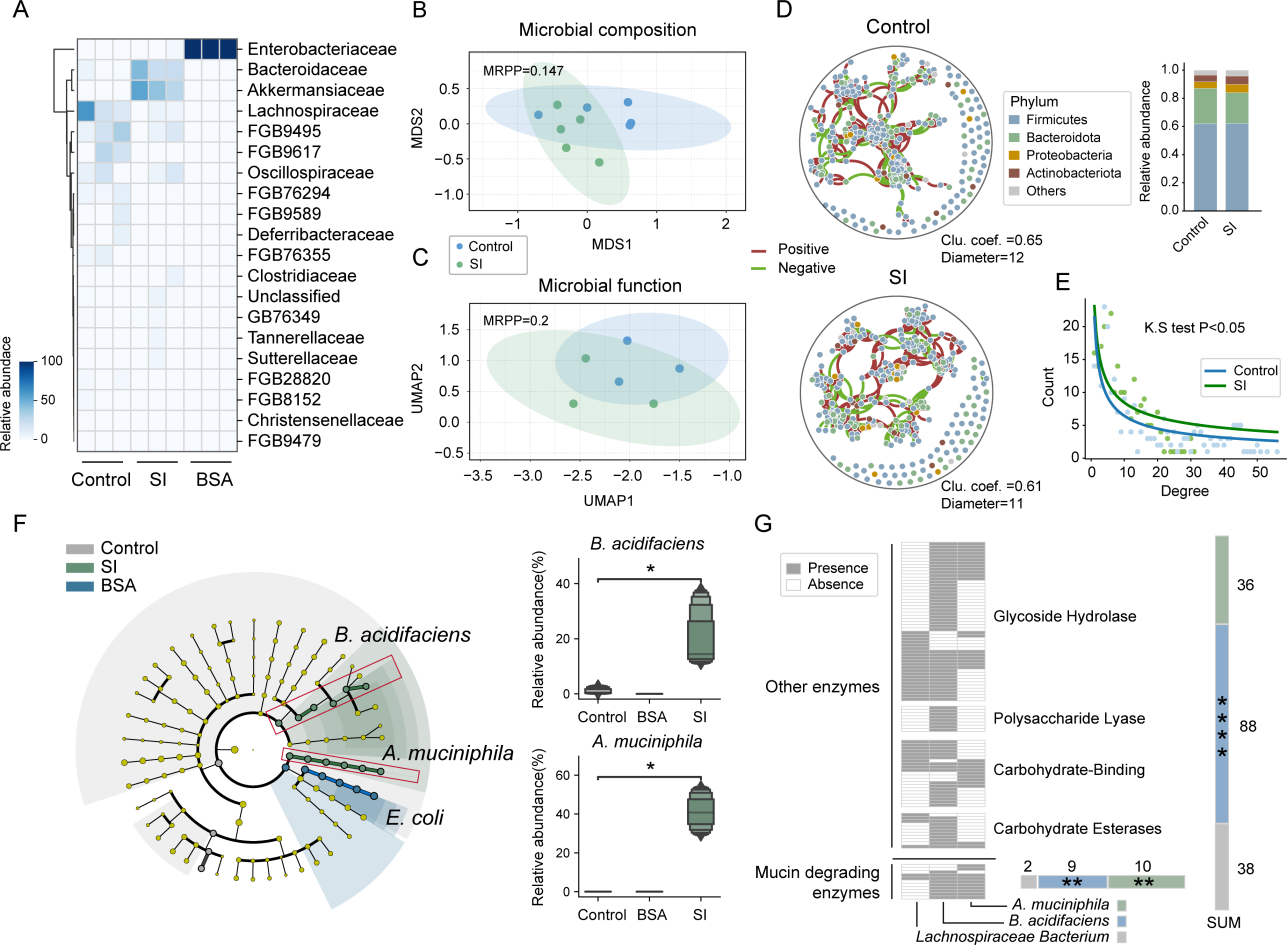
Specific gut microbes corresponded to sialidase inhibitor intervention in mice. (**A**) The heatmap figure shows the composition of gut microbiota in different groups at the family level. Microbial composition with NMDS (**B**) and microbial function with UMAP (**C**) in two groups (Control and SI). (**D**) The co-occurrence networks of the two groups are established. Different colors represent different phyla. The edges with red and green represent the positive and negative correlation, respectively. (**E**) The distribution of the vertex degree in two groups, and the Kolmogorov-Smirnov (K.S) test is used to estimate the identity of two distributions. (**F**) Left: the clade-specific taxonomic biomarkers of the gut microbiota in three groups (Control, SI, and BSA). The biomarkers at the species level are labeled. Right: the relative abundances of two species detected as biomarkers are shown (*P*-value < 0.05, Mann-Whitney and Wilcoxon test). (**G**) The presence of carbohydrate enzyme genes in three types of bacteria. (**P-value < 0.01; ****P-value < 0.0001, Two-sample *z*-test, two-tailed.)

The diverse microbial communities within the intestinal tract are also paramount to gut health (42). The use of antibiotics will result in dysbacteriosis, leading to a reduction in microbial diversity. *Escherichia coli*, a member of the Enterobacteriaceae family, has been observed to exhibit wide antibiotic resistance (43, 44), and after using the BSA, it has become the predominant microbial species within the intestinal tract in our study (Fig. 4A; Fig. S6). In contrast, the use of SI did not result in the destruction of the diversity in the gut microbiota (Fig. S7). The community structure and functional gene composition of the gut microbiota in the SI group remained similar to those in the Control group (Fig. 4B and C). Moreover, the construction of co-occurrence networks in different groups has demonstrated that the SI intervention exerted little influence on the symbiotic relationships of microbial communities within the intestinal tract (Fig. 4D). The SI intervention only resulted in limited alterations to the microbial symbiotic relationship. The network size (diameter 12:11), cluster coefficient (0.65:0.61), and network composition of the two groups were almost unchanged (Fig. 4D). Furthermore, the degree distribution of the co-occurrence network remained consistent with a scale-free network and followed a power-law distribution after SI treatment. This indicated that the intestinal microbial system retained a high level of resilience (Kolmogorov-Smirnov test, *P*-value < 0.05, Fig. 4E).

Notably, the relative abundance of *Akkermansia muciniphila* and *Bacteroides acidifaciens* was significantly increased after the intervention of SI (Fig. 4F), and these species have been reported to play a role in maintaining mucosal health by participating in the glycosylation of intestinal mucosa (45). To ascertain the potential causes of this microbial proliferation, we analyzed the carbohydrate gene profile data for *A. muciniphila* and *B. acidifaciens*, as well as the displaced microbe (*Lachnospiraceae bacterium*) from the Control group, retrieved from the Unified Human Gastrointestinal Genome (UHGG) database (46). The results demonstrated that the gene spectrum of mucopolysaccharide hydrolase in *A. muciniphila* and *B. acidifaciens* was significantly higher than that of the *Lachnospiraceae bacterium* (Fig. 4G, *P*-value < 0.01). Among these, *B. acidifaciens* exhibited the most extensive spectrum of carbohydrate metabolism (*P*-value < 0.0001), thus presenting an advantage in niche competition with other microbes due to its broad spectrum of carbon source uptake. In addition, *in vitro* culture of both microbes demonstrated that their growth was not affected when multiple carbon sources were present, and SI was added (Fig. S8). This provided a rationale for the enrichment of *B. acidifaciens* in the SI group based on the random clinical trial (Fig. 2F).

## DISCUSSION

Here, we investigated the effects of the SI in patients with mild-to-moderate UC. Our findings demonstrated that SI not only alleviated symptoms but also modulated the gut microbiome and improved intestinal health. Oseltamivir is one type of Food and Drug Administration (FDA) approved sialidase inhibitor (47). A recent study demonstrated that the control of sialic acid metabolism with oseltamivir can alleviate colitis in zebrafish (47). In our study, the notable improvements in abdominal pain and total symptom scores observed after SI intervention compared to the control group provided evidence supporting the therapeutic efficacy of SI in UC management. Furthermore, the endoscopic remission achieved after 1 month of SI intervention, along with significantly reduced DUBLIN, reinforced its clinical utility.

In addition, we explored the metabolic changes associated with SI intervention. Utilizing untargeted metabolomics, we found that SI induced alterations in the composition of fecal metabolites. These metabolites were enriched in pathways related to arginine biosynthesis, glutathione metabolism, and nitrogen metabolism. The role of arginine in gut health has been previously highlighted, with studies indicating its involvement in regulating intestinal barrier function and inflammatory responses (48–50). Glutathione metabolism has also been reported to play an important role in immune regulation (51). Beyond pathway-level enrichment, several key metabolites were specifically altered after SI intervention. Among them, urea was significantly increased, whereas L-glutamate, N-acetylornithine, and 5-oxoproline were significantly decreased. These changes suggest that SI intervention was associated with remodeling of amino acid metabolism, nitrogen metabolism, and glutathione-related metabolic processes in the gut (52, 53). Notably, the reduction in 5-oxoproline, a metabolite involved in glutathione cycling, may be consistent with altered glutathione metabolism (54). Meanwhile, the decreases in L-glutamate and N-acetylornithine, together with the increase in urea, suggest a shift in intestinal nitrogen metabolic output after SI treatment. It suggested that SI may enhance the intestinal metabolic environment and promote processes that are beneficial for mucosal health and inflammation control.

Moreover, our analysis revealed a significant correlation between the altered metabolic landscape and the gut microbiota composition. We identified several beneficial bacteria enriched after SI intervention, especially bacteria producing butyrate such as *Bacteroides* (20), *Romboutsia* (55), *Anaerostipes* (56), *Flavonifractor* (57), and *Butyricicoccus* (58). This was consistent with existing literature that highlights the role of butyrate in maintaining intestinal health by supporting epithelial integrity and exerting anti-inflammatory effects (59). The correlation between SI intervention and increased butyrate-producing bacteria suggested a potential mechanism by which SI enriched the favorable gut microbiota, contributing to the therapeutic effects observed in UC patients.

Furthermore, we conducted experiments in DSS-induced colitis mouse models to further elucidate the mechanism by which SI exerts its protective effects. Our results demonstrated that SI mitigated DSS-induced inflammation, as evidenced by reduced DAI scores and preservation of colon length. SI also significantly reduced the expression of inflammatory markers, including *Tnf-*α, *IL-6*, and *IL-1*β. This aligns with findings from previous studies that the increase in intestinal bacterial sialidase activity can aggravate the severity of acute DSS-induced colitis in mice (60). In addition, our research suggested that SI helped restore the balance of the gut microbiota, thereby reducing microbial-mediated inflammation. The integrity of the intestinal mucus layer, which plays a crucial role in preventing pathogen invasion, was also improved with SI intervention. The increased mucus layer thickness and normalized expression of MUC2 and other mucus-related genes, such as *Agr2* and *Ern2* (61), indicate that SI not only enhances mucosal barrier function but also promotes a favorable environment for beneficial microbes.

Our findings align with other studies highlighting the importance of the intestinal mucus layer in maintaining gut health and preventing inflammation (3). Previous research has shown that a robust mucus layer is essential for preventing bacterial translocation and protecting against colitis (62). The increased thickness of the mucus layer in the SI group suggested enhanced protection against harmful microbes. In this process, we found that *A. muciniphila* and *B. acidifaciens* were enriched. Additionally, we found that SI did not significantly disrupt the symbiotic relationships within the gut microbiota, maintaining a high level of resilience. Although both BSA and SI partially restored the expression of certain mucus-related genes, their overall effects on colitis were markedly different. SI provided broader protection against DSS-induced colitis, including improvement in colon length, histopathological injury, mucus layer thickness, and inflammatory status, while also preserving microbial community structure. In contrast, BSA did not confer comparable overall protection and was associated with disruption of the gut microbiota. These findings suggest that restoration of individual mucus-related genes alone is insufficient to indicate therapeutic equivalence and that SI may offer a more balanced strategy for mucosal protection by limiting mucus degradation while preserving microbial homeostasis. These findings underscore the potential of SI to maintain microbial diversity while enriching beneficial taxa, which is critical for gut homeostasis. Rather than exerting broad antimicrobial pressure, SI may function as an ecological filter within the mucus niche. By restricting terminal sialic acid release from mucins, SI is likely to reduce the competitive advantage of microbes that rely heavily on the initial desialylation step, while favoring mucin-associated commensals with greater glycan-utilization flexibility and higher ecological fitness in a nutrient-diverse colonic environment. In this context, the enrichment of *A. muciniphila* and *B. acidifaciens* in our study may reflect not only their broader CAZyme repertoire but also their compatibility with a barrier-supportive intestinal microenvironment. Several studies have emphasized that effective intestinal intervention depends on coordinated restoration of the intestinal microenvironment, including modulation of microbiota and metabolites, reinforcement of mucus secretion and tight junctions, and attenuation of oxidative and inflammatory injury. Therefore, the selectivity observed after SI treatment may be better understood as ecological filtering coupled with barrier restoration, rather than simple survival through one or a few alternative glycoside hydrolases alone (63–65).

A recent study reported that polyvalent sialidase inhibitors showed strong *in vitro* inhibition of bacterial sialidases and suppressed mucin desialylation but did not produce robust improvement in disease activity in a murine colitis model (66). These findings do not necessarily conflict with ours but instead suggest that the *in vivo* efficacy of sialidase-targeted therapy may depend on the inhibitor scaffold, target spectrum, timing of intervention, and disease model. In our study, SI was administered before DSS exposure rather than simultaneously with DSS, which may have allowed modulation of the mucus barrier and gut microbial environment prior to inflammatory injury. Oseltamivir was associated with improvement in clinical and experimental indices, mucus barrier integrity, and microbiota-related features, supporting the potential of this approach while also underscoring the need for further mechanistic and comparative studies.

Sialidase, as a kind of mucinase, is a factor leading to the thinning of the mucus layer in patients with UC (67). The release of sialic acid by sialidase at the end of the host MUC2 is an important nutrient source for many enteric commensal and pathogenic bacteria (68). However, some specific gut microbiota (e.g., *A. muciniphila* and *B. acidifaciens*) can express sialidase to release sialic acid from MUC2 glycoprotein (45). Although the sialidase was inhibited after SI intervention, there were more than 10 other glycoside hydrolases, including sialidase and fucosidase (45). Our additional *in vitro* experiments suggest that SI does not directly inhibit the growth of *A. muciniphila* or *B. acidifaciens* in nutrient-rich conditions, but it does impair their growth when mucin is the sole carbon source. Therefore, the enrichment of these species *in vivo* is unlikely to be explained simply by unaffected mucin utilization under SI treatment alone. Instead, their persistence may reflect greater metabolic flexibility and broader substrate-utilization capacity in the complex intestinal environment, which is consistent with their richer glycoside hydrolase repertoires. They are mucolytic bacteria, which also promote mucin secretion in the host, suggesting that they can participate in mucus turnover (45).

While this study provides preliminary evidence for the potential value of SI as an adjunctive strategy for UC, several limitations should be acknowledged. Although the improvement observed in the SI group supports the rationale for further investigation, this pilot randomized trial involved a relatively small sample size and should be considered an exploratory study. Since all patients received mesalazine as background therapy, individual variability in mesalazine responsiveness cannot be fully excluded. In addition, although SI intervention increased colonic mucus layer thickness in the mouse model, the colonic concentration of SI was not measured in this study. The 4-week oseltamivir regimen was exploratory and based on existing human safety experience. Viral sequencing was not performed because the study focused on gut sialidase-related mechanisms rather than antiviral effects. Moreover, in the animal study, SI was administered before DSS exposure to reduce potential interference between SI and DSS; hence, this design primarily supports a protective rather than therapeutic effect. Future studies with larger cohorts, pharmacokinetic analysis, and post-induction therapeutic models are warranted.

In conclusion, our study demonstrates that SI intervention effectively alleviates symptoms and improves intestinal health in mild-to-moderate UC by modulating the gut microbiome and metabolic pathways. SI enriched butyrate-producing bacteria, enhanced mucosal barrier function, and reduced inflammation, as evidenced by improved clinical scores, endoscopic remission, and reduced inflammatory markers in both patients and DSS-induced colitis models. The enrichment of beneficial mucolytic bacteria, such as *A. muciniphila* and *B. acidifaciens*, further supports SI’s role in maintaining gut homeostasis. These findings highlight SI as a promising therapeutic strategy for UC, targeting sialidase activity to restore gut health and mitigate inflammation. Further studies are warranted to validate its long-term efficacy and safety.

## Data Availability

A STORMS (Strengthening The Organizing and Reporting of Microbiome Studies) checklist is available at https://doi.org/10.5281/zenodo.20051216. The raw sequence data reported in this paper have been deposited in the Genome Sequence Archive in National Genomics Data Center, China National Center for Bioinformation/Beijing Institute of Genomics, Chinese Academy of Sciences, under accession numbers CRA019765 and CRA019772 and are publicly accessible at https://bigd.big.ac.cn/gsa.

## SUPPLEMENTAL MATERIAL

Supplemental material msystems.00194-26-s0001.pdf Additional experimental details and supplemental figures and tables.

ASM does not own the copyrights to Supplemental Material that may be linked to, or accessed through, an article. The authors have granted ASM a non-exclusive, world-wide license to publish the Supplemental Material files. Please contact the corresponding author directly for reuse.
