# Supplementary material for: Sialidase inhibitor modulates gut microbiota and enhances mucosal protection in the treatment of ulcerative colitis
Source: mSystems. 2026 Jul 10;11(8):e00194-26. doi: 10.1128/msystems.00194-26 (PMC13483315; doi:10.1128/msystems.00194-26)
Supplement: Supplementary file 1 [file msystems.00194-26-s0001.pdf]

# Supplementary Methods

## Real-Time reverse transcription polymerase chain reaction

The distal colon tissues frozen in the -80 °C were taken out and added with Trizol reagent (SK1312; Sango, Shanghai, China) to extract the total RNA according to the manufacturer's instructions. RNA concentration was calculated by measuring absorbance at 260 nm using a microplate reader and NanoDrop 2000 software. The RNA was reverse transcribed into cDNA by the AMV First Strand cDNA Synthesis kit (SK2445; Sango, Shanghai, China). The Real-time PCR was conducted by using Takara SybrGreen PCR Master Mix (RR820A; Takara, Kyoto, Japan) on CFX Connect Real-time PCR System (BIO-RAD, California, USA). The primer sequences were shown below. The quantitative expression levels of each target gene *(TNF-α, IL-6, IL-1β, Muc2, Tff3, Ern2, Agr2*) were analyzed by the ΔΔCT method and normalized to that of GAPDH.

Real-time reverse transcription polymerase chain reaction primer sequences

| Gene | Forward (5'–3') | Reverse (5'–3') |
|---|---|---|
| *TNF-α* | GCCATGCTGTATGGAGTCTCG | CCACTTCCGTTCTGAACCTTG |
| *IL-6* | CTGCAAGAGACTTCCATCCAG | AGTGGTATAGACAGGTCTGTTGG |
| *IL-1β* | GCAACTGTTCCTGAACTCAACT | ATCTTTTGGGGTCCGTCAACT |
| *Muc2* | CAACAACCAGCTCATCTCC | GTGATGGAGCCTGAAACAC |
| *Tff3* | CTAATGCTGTTGGTGGTCC | CATACATTGGCTTGGAGACAG |
| *Ern2* | CAATGTATGTCACAGAAATGGC | CCTGTTGTTTCTGGGTTCC |
| *Agr2* | CCTCAGACACTCTCCAGAG | GGTCTGTTGCTTGTCTTGG |

## Growth of *Bacteroides acidifaciens* and *Akkermansia muciniphila* in vitro

*Bacteroides acidifaciens* (BNCC353574) and *Akkermansia muciniphila* (BNCC341917) were purchased from BeNa Culture Collection (BNCC, China) and activated according to the instructions. To study the growth of these microbes with rich and single carbon sources, we referenced the culture medium in Ryan et al. (1) and used the same carbon source ratio for 36 hours, measuring OD600 every 6 hours. In addition, for the culture medium with added SI, we referred to Quosdorf et al. (2), adding oseltamivir carboxylate to the culture medium and controlling the SI concentration in the culture medium to 200 uM.

## Reference

1. Ryan D, Bornet E, Prezza G, Alampalli SV, Franco de Carvalho T, Felchle H, Ebbecke T, Hayward RJ, Deutschbauer AM, Barquist L. 2024. An expanded transcriptome atlas for Bacteroides thetaiotaomicron reveals a small RNA that modulates tetracycline sensitivity. Nature Microbiology 9:1130-1144.
2. Quosdorf S, Schuetz A, Kolodziej H. 2017. Different inhibitory potencies of oseltamivir carboxylate, zanamivir, and several tannins on bacterial and viral neuraminidases as assessed in a cell-free fluorescence-based enzyme inhibition assay. Molecules 22:1989.

# Supplementary Tables

**Table S1.** Summary descriptives table of the patients

| | Non-SI | SI | P-value |
|---|---|---|---|
| BMI | 21.0 (±2.51) | 23.1 (±3.32) | 0.154 |
| No Drink | 9 (100%) | 10 (100%) | |
| Smoke: | | | 0.474 |
| No | 8 (88.9%) | 10 (100%) | |
| Yes | 1 (11.1%) | 0 (0.00%) | |
| Age | 41.3 (±16.5) | 44.1 (±19.9) | 0.715 |
| Gender: | | | 0.656 |
| Female | 5 (55.6%) | 4 (40.0%) | |
| Male | 4 (44.4%) | 6 (60.0%) | |
| Montreal type: | | | 0.607 |
| E1 | 3 (33.3%) | 5 (50.0%) | |
| E2 | 3 (33.3%) | 1 (10.0%) | |
| E3 | 3 (33.3%) | 4 (40.0%) | |
| DUBLIN before intervention | 4.56 (±2.18) | 5.80 (±2.57) | 0.312 |
| MMES before intervention | 10.0 (±2.50) | 10.85 (±3.19) | 0.523 |

For categorical variables: Sample size (percentage); for continuous variables: mean (±standard deviation).

**Table S2.** The symptom score in different groups

| | Non-SI | SI | P-value |
|---|---|---|---|
| Reduction of total symptom score | 0. 79±1.672 | 2.29±0.699 | 0.01 |
| Reduction of stomachache score | 0.21±0.699 | 0.79±0.579 | 0.027 |
| Reduction of diarrhea score | 0.21±0.975 | 0.79±0.699 | 0.086 |
| Reduction of hematochezia score | 0.36±0.497 | 0.71±0.611 | 0.102 |

Value: mean (±standard deviation).

**Table S3.** Adverse events

| Metabolites | P-value | Log2 Fold Change (SI vs. Non-SI) | VIP | Pathway |
|---|---|---|---|---|
| L-Glutamate | 0.030 | -1.207 | 1.931 | Arginine biosynthesis; Glutathione metabolism; Nitrogen metabolism |
| N-Acetylornithine | 0.047 | -2.168 | 1.571 | Arginine biosynthesis |
| Urea | 0.018 | 4.239 | 1.482 | Arginine biosynthesis |
| 5-Oxoproline | 0.030 | -0.872 | 1.881 | Glutathione metabolism |

**Table S4.** Differential metabolites in enriched pathways

| | No./total No. (%) | | |
|---|---|---|---|
| | Non-SI | SI | P-value |
| New gastrointestinal bleeding | 2/9(20) | 3/10(20) | 0.70 |
| New joint pain | 1/9(0) | 1/10(0) | 0.94 |
| Systemic pain | 1/9(0) | 1/10(0) | 0.94 |
| Skin rash | 0/9(0) | 1/10(0) | 0.33 |
| Arrhythmia | 0/9(0) | 1/10(0) | 0.33 |

## Supplementary Figures

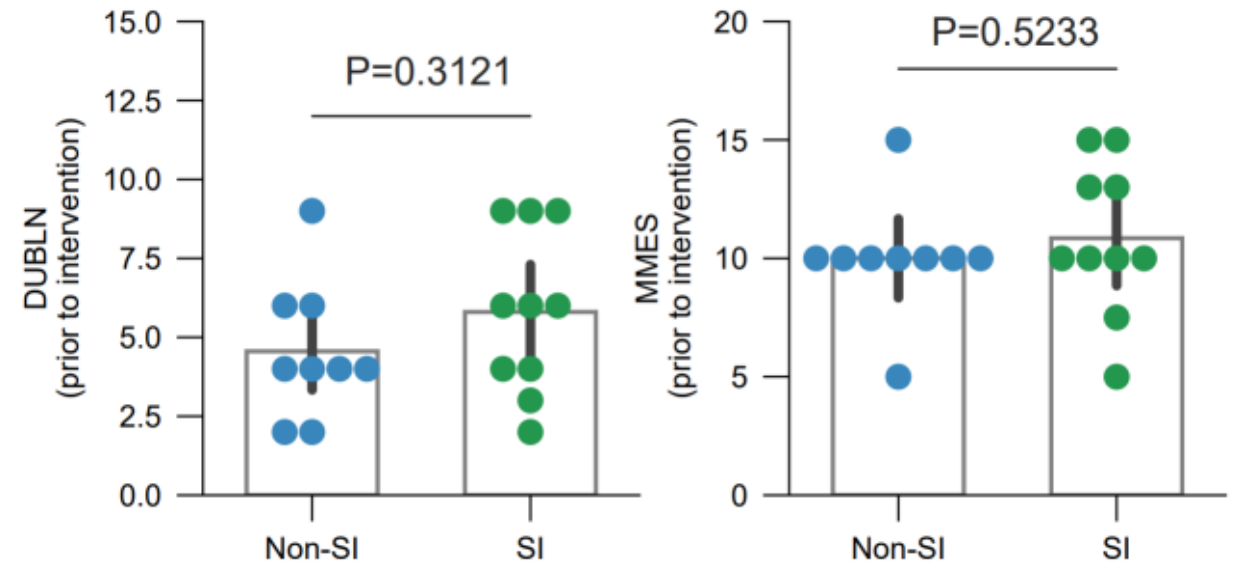


**Figure S1**. From left to right: DUBLIN and Modified Mayo Endoscopic Score (MMES) for patients in the non-SI and SI groups, respectively using Mann-Whitney-Wilcoxon test.

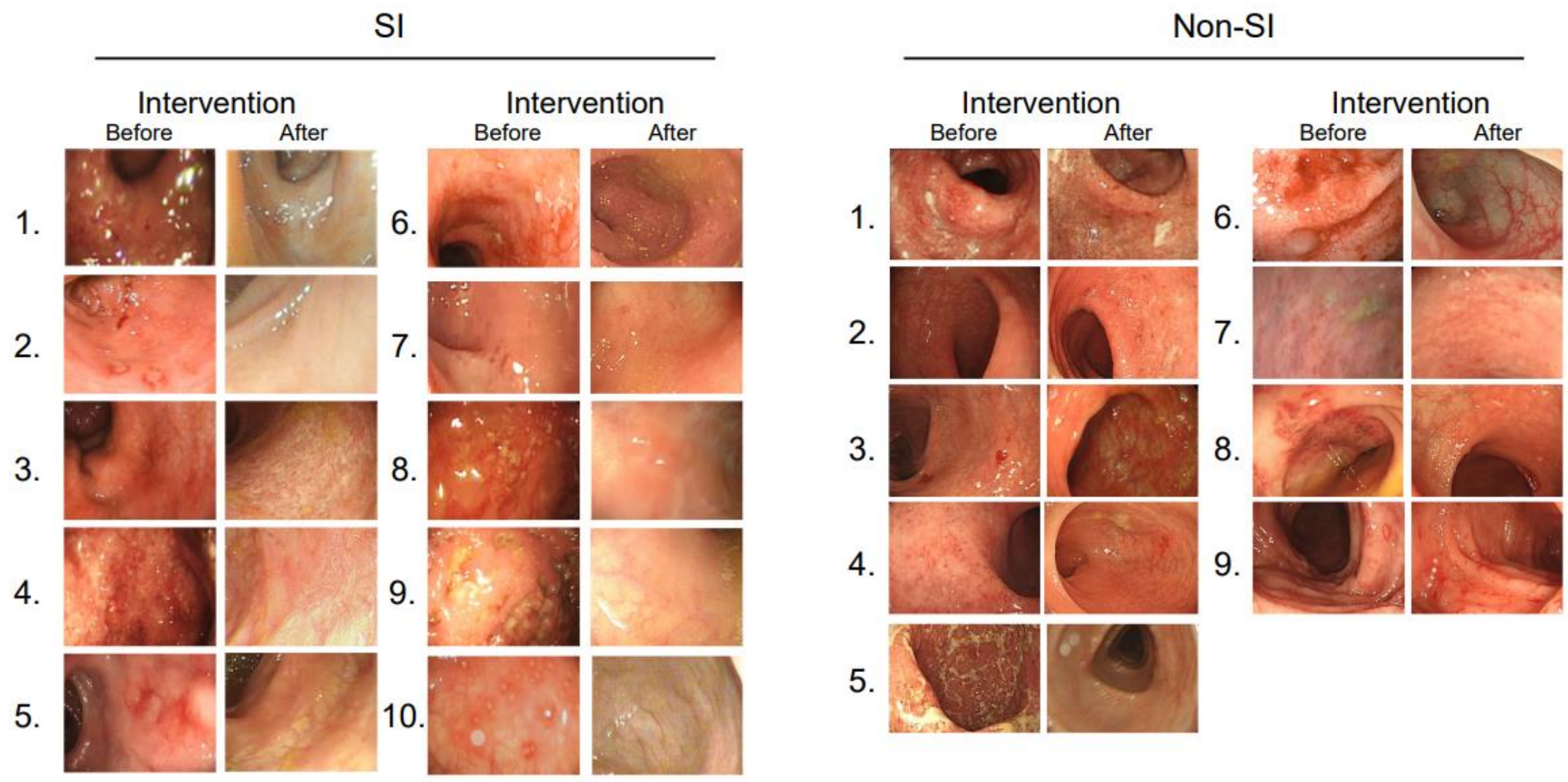


**Figure S2**. Representative images of the intestines for each patient in two groups.

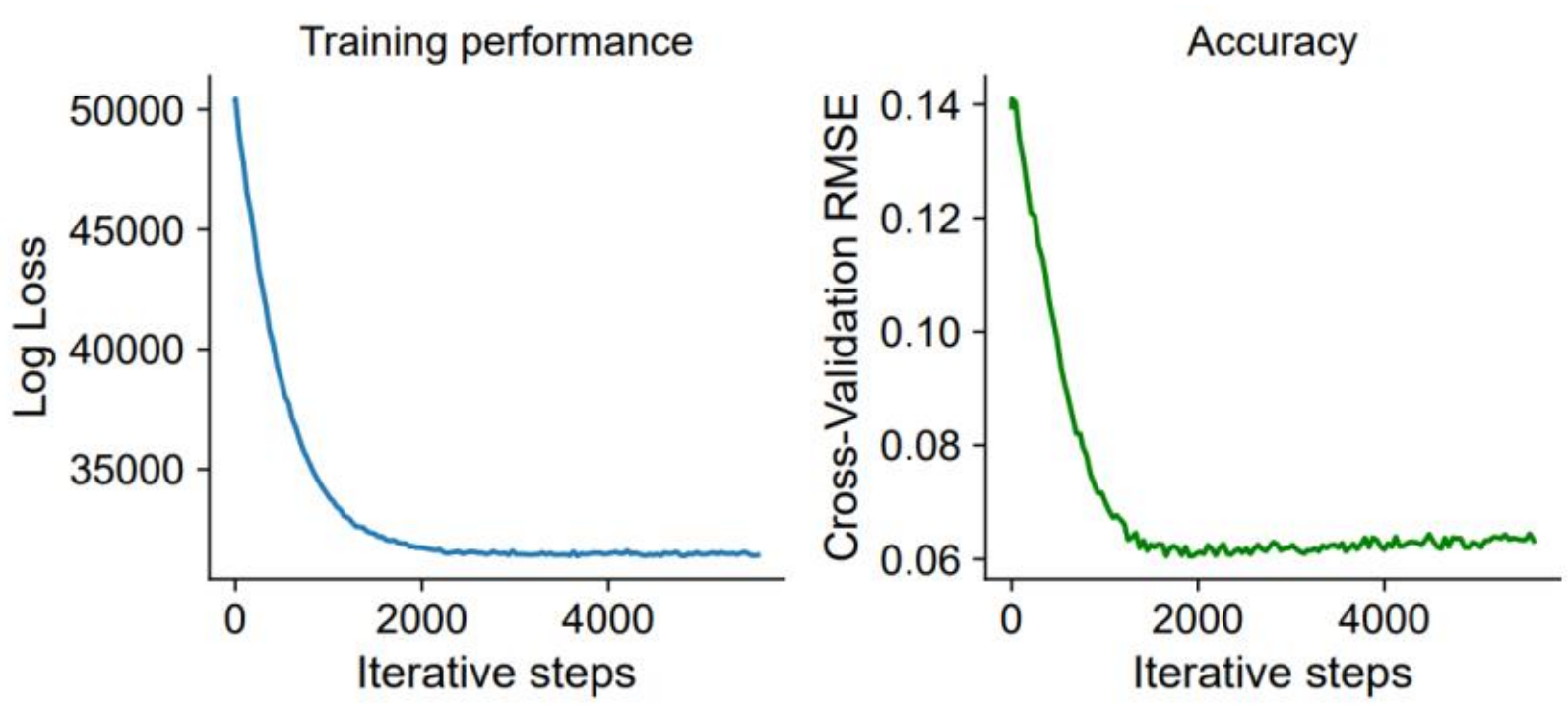


**Figure S3.** From left to right, the loss size and root-mean-square error (RMSE) are shown to estimate the accuracy of the deep-learning model.

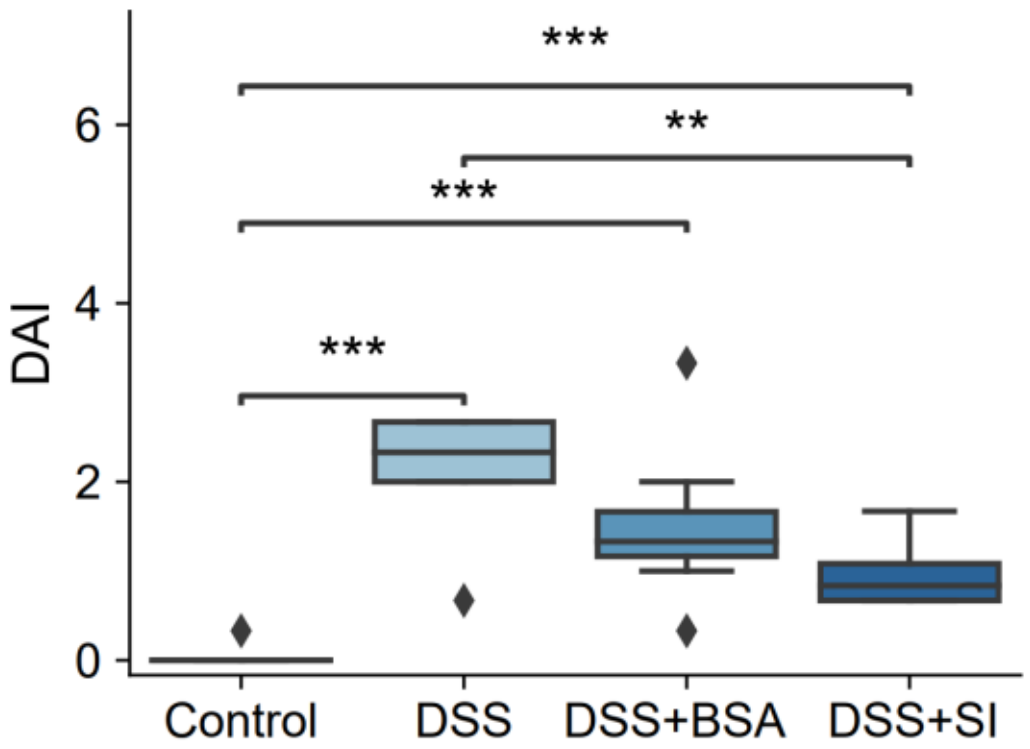


**Figure S4.** The disease activity index (DAI) in different groups. (** P-value < 0.01; *** P-value < 0.001, **** P-value < 0.0001, Mann-Whitney-Wilcoxon test)

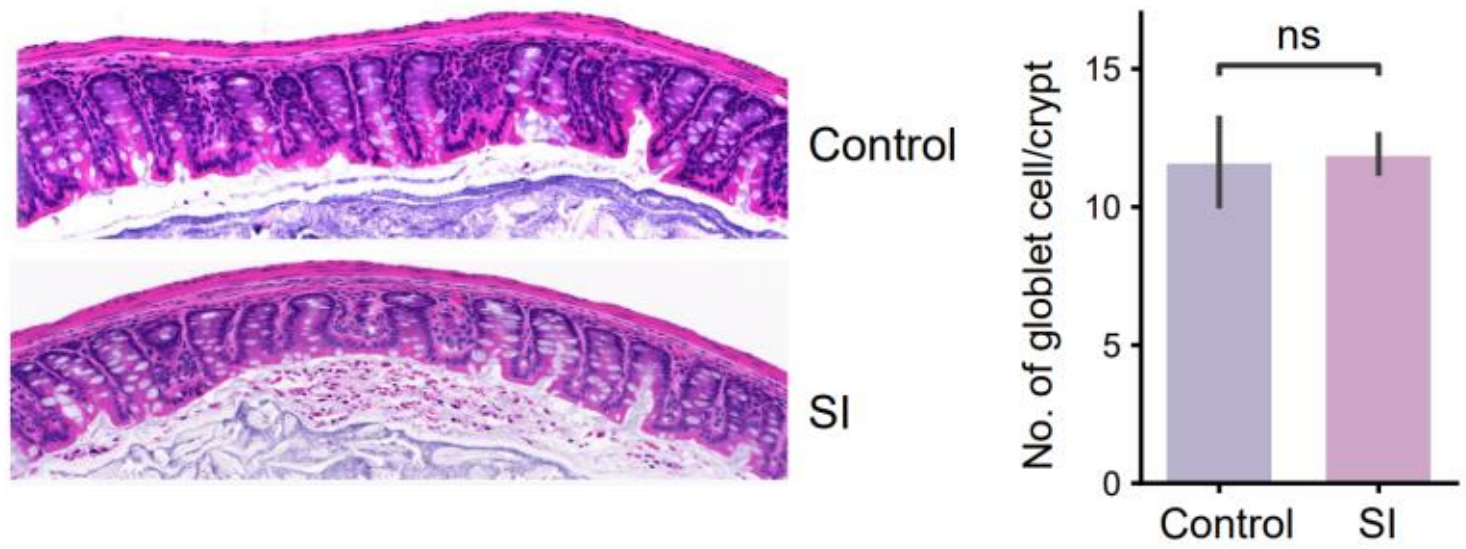


**Figure S5.** Left: Hematoxylin-eosin (HE) staining (magnification 20×) of the colon tissues. Right: Numbers of goblet cell per crypt. (ns: P-value > 0.05, Mann-Whitney-Wilcoxon test)

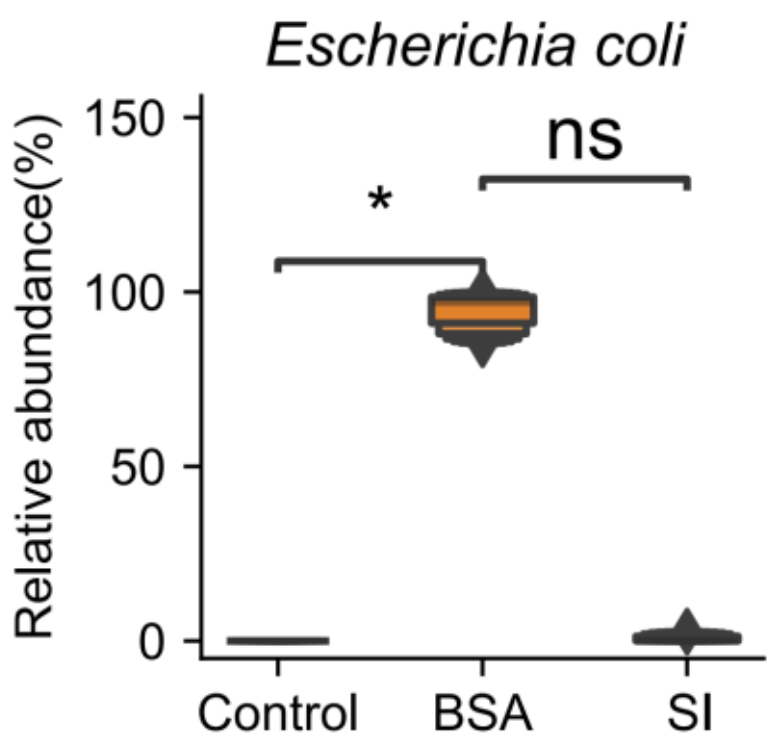


**Figure S6.** The relative abundance of *Escherichia coli* in different groups using metagenomic sequencing. (* P-value < 0.05; ns: P-value > 0.05, Mann-Whitney-Wilcoxon test)

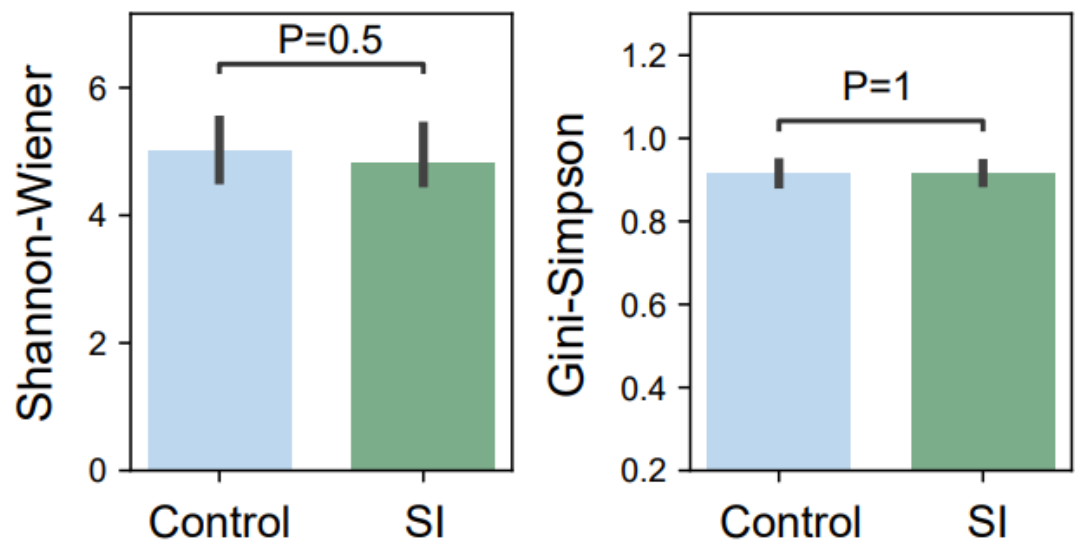


**Figure S7.** The Shannon-Wiener and Gini-Simpson index are calculated to estimate the alpha-diversity, respectively (Mann-Whitney-Wilcoxon test).

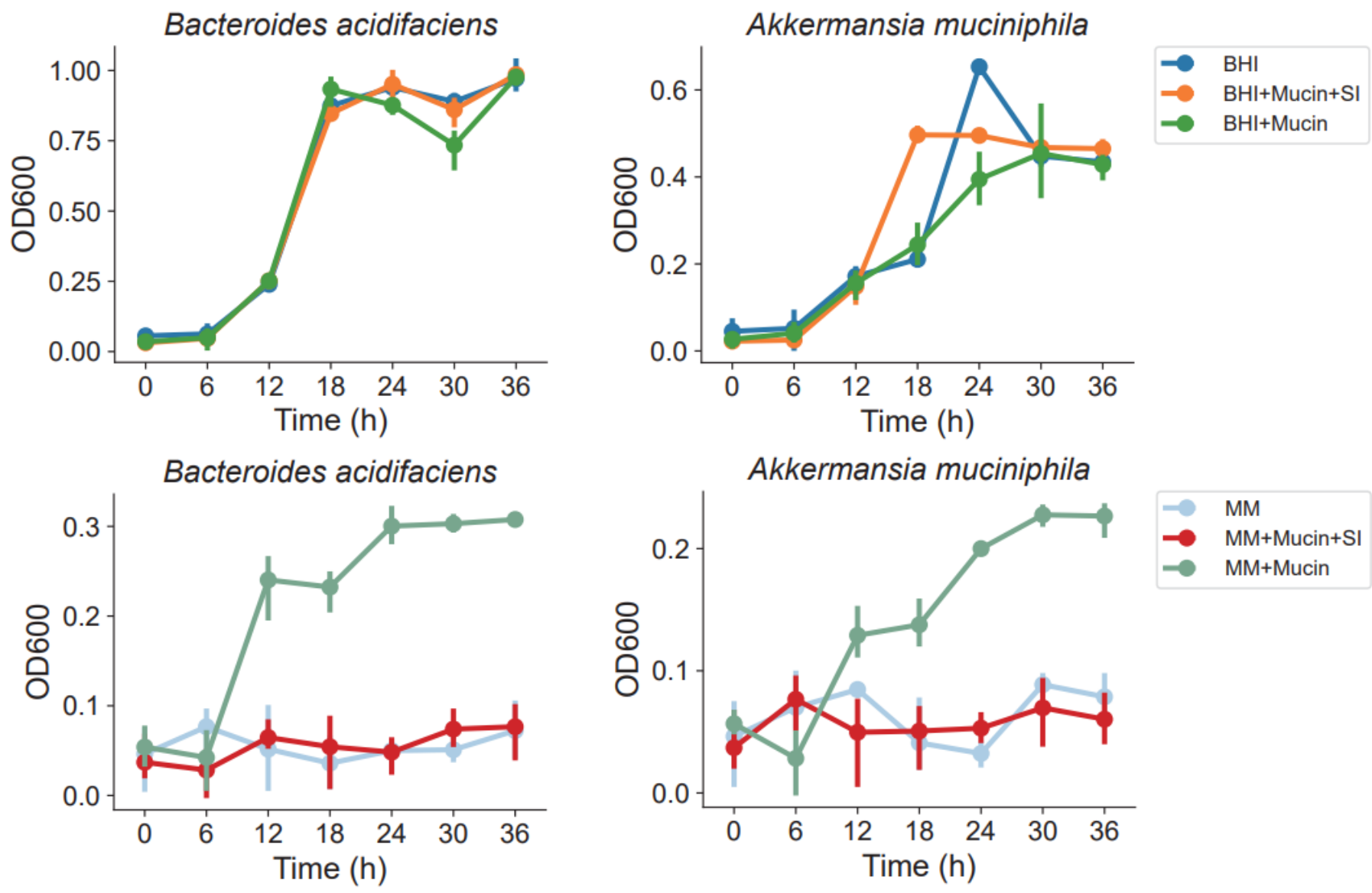


**Figure S8.** Growth curves of *Bacteroides acidifaciens* and *Akkermansia muciniphila* under different culture conditions.
